# Growth, Properties, and Applications of Branched Carbon Nanostructures

**DOI:** 10.3390/nano11102728

**Published:** 2021-10-15

**Authors:** Sharali Malik, Silvia Marchesan

**Affiliations:** 1 Karlsruhe Institute of Technology, Institute of Quantum Materials and Technology, Hermann-von-Helmholtz-Platz 1, 76131 Karlsruhe, Germany; 2 Department of Chemical and Pharmaceutical Sciences, University of Trieste, Via L. Giorgieri 1, 34127 Trieste, Italy; smarchesan@units.it

**Keywords:** carbon nanotubes, carbon nanofibers, carbon nanostructures, graphene nanosheets, catalysis, energy storage, sensors, tissue engineering, nanocomposites, COST Action EsSENce CA19118

## Abstract

Nanomaterials featuring branched carbon nanotubes (b-CNTs), nanofibers (b-CNFs), or other types of carbon nanostructures (CNSs) are of great interest due to their outstanding mechanical and electronic properties. They are promising components of nanodevices for a wide variety of advanced applications spanning from batteries and fuel cells to conductive-tissue regeneration in medicine. In this concise review, we describe the methods to produce branched CNSs, with particular emphasis on the most widely used b-CNTs, the experimental and theoretical studies on their properties, and the wide range of demonstrated and proposed applications, highlighting the branching structural features that ultimately allow for enhanced performance relative to traditional, unbranched CNSs.

## 1. Introduction

The late Stone Age, known as the Neolithic Age (10,000–4500 BC) marked the first extensive use of composite materials in construction—the “mudbrick”. The beginnings of an agricultural system also trace back to this period. By-products of agriculture, such as straw, were used as reinforcing materials in these mudbricks, thus providing an early example of “recycling”. Mudbricks can therefore be considered as the first recorded artifact containing a branched material as a matrix reinforcing component [1].

Many other biological systems have branched structures where their functional morphology is key to their advanced functionality. For example, the correlation of branching in plants with respect to their functional morphology and mechanical behavior have led to concepts applicable in synthetic branched-fiber materials [2], in the bio-inspired design of polymer nanocomposites [3], in tissue engineering [4], and in superhydrophobicity—the so-called “lotus leaf” effect [5]. Currently, branched carbon nanotubes (b-CNTs) and branched carbon nanofibers (b-CNFs) are of great technological interest due to their electronic and mechanical properties. Although less common, branched CNTs can also be grown onto graphene-based materials, such as reduced graphene oxide (rGO), in a combination of 1D and 2D components to attain 3D hierarchical architectures [6]. All these materials can form 2D and 3D ordered networks, and the aim of this mini-review is to highlight some of their proof-of-principle and proposed applications.

## 2. Branching and Growth of Carbon Nanostructures (CNSs)

### 2.1. Experimental Studies and Branched CNS Production

Currently, Ni, Co, Fe, and Cu or their alloys are used as catalyst particles to decorate CNFs or CNTs to form branches. The main but by no means only used [7] growth method is some form of chemical vapor deposition (CVD) process [8], which can be further modified, for instance through plasma enhancement, to increase the geometrical diversity of the resulting nanomaterials [9,10,11]. Earlier, Terrones et al. found that sulfur plays an important role in the formation of branched nanotube networks with stacked-cone morphologies [12]. In particular, sulfur acts as a CNT branching promoter, as its interaction with the C atoms favors the formation of non-hexagonal rings in the sp^2^ lattice, thus introducing a negative curvature in the case of heptagons (leading to branch opening) or a positive curvature in the case of pentagons (leading to tip closing) [12]. More recently, Huang et al. fabricated branched CNTs by a CVD process in the presence of thiols. They concluded that “sulfur in thiol may reduce the melting point of iron (Fe: 1535 °C, FeS: 1193 °C) during the pyrolysis process. This will result in the enlargement of iron particles which is responsible for nanotube growth”. This finding is important, as they noted that these larger particles were more likely to split into smaller ones, leading to branched nanotube growth [13].

Most researchers who do not fabricate their own b-CNTs use the so-called CNSs supplied by Applied Nanostructured Solutions LLC (Lockheed Martin Corporation, Bethesda, MD, USA) [14]. The CNSs are fabricated in a CVD process on a moving substrate (e.g., glass fiber) with a growth rate of up to several microns per second. The process uses an iron catalyst, which initializes CNT growth, and then splits, forming Y-junction CNTs. The CNTs within the CNS have a more defective structure than those in a conventional CNT bundle. The CNS bundles consist of aligned CNTs whose inner walls are intact, and the outer walls have defects with 5 or 7 membered carbon rings, which covalently bind to adjacent CNTs. On average, the bundles are 70 μm long and 10 μm thick with multi-wall CNTs (MWCNTs) of ca. 9 nm diameter. “These defective features are characterized by its highly entangled, branched, cross-linked, and wall-sharing architecture” [15].

**Figure 1:**
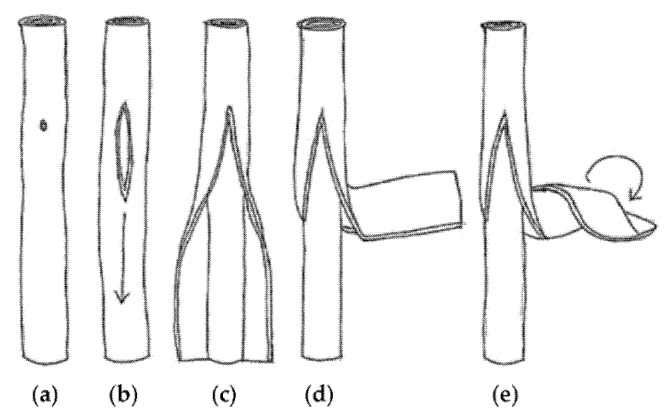
A schematic diagram of the suggested “unzipping” and “re-rolling” sequence: (**a**) formation of unzipping point; (**b**) onset of unzipping; (**c**) unzipping and onset of peeling of inner parallel tubes; (**d**) outer layers peeling out as a sheet; (**e**) onset of re-rolling of outer layers. Reproduced from [16].

**Figure 2:**
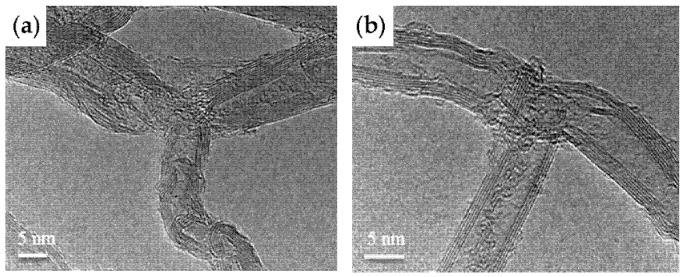
High-resolution transmission electron microscopy (HR-TEM) micrographs of (**a**) Y-junction MWCNTs; (**b**) T-junction MWCNTs. Reproduced from [16].

Malik et al. fabricated branched MWCNTs using commercially available MWCNTs (Baytubes© from Bayer Material Science A.G., Leverkusen, Germany) by introducing defects in the outer tubes, which split and re-rolled to form branched MWCNTs (Figure 1) with Y-junctions and T-junctions (Figure 2) [16]. They also found that if they used the same process on thinner MWCNTs, such as triple-walled MWCNTs, then tri-layer graphene ribbons formed. Tri-layer graphene is currently an interesting quantum material [17]. The indications are that only a few outer walls of the branched MWCNTs are defective; nevertheless, enhanced properties emerge, such as a lower percolation threshold. The inner tubes of the branched MWCNTs remain intact and so retain their native electrical and mechanical properties.

A simple, one-step co-pyrolysis method was developed to produce branched CNTs with controlled N-doping at the junctions, using hexamethylenetetramine and benzene as nitrogen and carbon sources. The catalyst splitting at the tips of the CNTs led to branching. Interestingly, it was found that the difference in the vapor pressure and the insolubility of the precursors were crucial aspects for the generation of the intratubular junctions connecting the b-CNTs [18].

Another type of approach to attain branched CNTs is based on the covalent or non-covalent functionalization with branched or dendritic structures to connect the CNTs with each other. The most commonly used procedures rely on grafting onto the CNTs with branched or dendritic polymers [19,20,21]. However, covalent functionalization is well-known to affect the electronic properties of CNSs and requires careful monitoring to preserve the CNS [22]. Alternatively, π–π interactions offer an efficient strategy to non-covalently attach CNTs to aromatic and multi-functional linkers, and, thus, to each other into branched structures. For instance, a molecular clip was designed displaying an open cavity with four anthracene panels that could capture single-walled carbon nanotubes (SWCNTs) to yield highly monodispersed and stable aqueous nanocarbon composites [23].

In recent years, environmental awareness has fostered increasing interest in the development of green production methods for carbon nanostructures [24] and their functionalization with virtually zero waste generation [25]. Diazonium salts have been proposed as covalent linkers for the tips of SWCNTs that were previously wrapped in DNA to protect the sidewalls from undesired functionalization, thus enabling the preferential crosslinking of the CNT ends in water and at room temperature [26]. Environmentally-friendly carbon sources, such as vegetable oils, have also been used to produce b-CNTs [27]. The upcycling of plastic waste for CNT production is another attractive approach to lower its impact on the environment [28] and was also applied to branched CNTs. In particular, Y-type branched MWCNTs were produced through the upcycling of polyethylene terephthalate (PET) waste, through a rotating cathode arc discharge technique. It was found that the soot obtained from the anode contained solid carbon spheres, which were formed at the lower temperature region of the anode (ca. 1700 °C), and which could be converted into long “Y” type branched and non-branched MWCNTs at approximately 2600 °C. Conversely, soot deposited on the cathode was composed of thinner MWCNTs and other nanoparticles (NPs), with the tubes generally featuring a higher graphitization degree, compared to those on the cathode [29].

### 2.2. Theoretical Studies

Theoretical research on branched CNSs has given greater insight into a number of parameters of interest, such as the type and nature of defects, for technological applications. Zang and Glukhova studied the formation of the T-shaped junction between SWCNTs. They combined a triangulated mesh with molecular dynamics (MDs) to allow for the generation of several topological configurations of the contact point between different carbon nanostructures, such as fullerenes, graphene, and CNTs, including T-, X-, and Y-type junctions with atomistic models [30]. Classical MD simulations were employed to enable better design of b-CNTs, and a comparison was made between the properties of b-CNTs with V-, T-, and Y-junctions and their effects on the performance of the resulting nanopins as part connectors in nanodevices [31]. DFT calculations were combined with the experimental bottom-up synthesis of a junction unit of b-CNTs to study its interesting optoelectronic properties [32].

The heatflow through b-CNTs with T-junctions was studied by atomistic models, with particular attention to the branch length and strain effects on the thermal transport. Remarkably, it was found that the heat flew straight, rather than sideways, inside the T-junction, using an asymmetric temperature setup. Such an observation is in disagreement with the conventional thermal circuit calculations, and may be explained through ballistic phonon transport, phonons with differing interactions or scattering with the defective atoms at the T-junction. Furthermore, the tensile strain proved to be a useful parameter to control the thermal transport, with potential implications for heat management uses [33]. Energy transmission is dependent on the type of junction too, as demonstrated in a detailed study on phonon scattering on T-type and X-type CNT junctions [34].

In silico studies of the mechanism of nanowelding a branched network of SWCNTs were performed for tissue engineering applications [4], whereby CNTs are very promising [35,36], especially to repair the bone [37,38] and conductive tissues [39], such as the nerve [40] and the heart [41]. Defective regions of SWCNTs were found to absorb more energy than defect-free regions, which acted as hot-spots for nanowelding and the formation of b-CNT networks [4]. Further studies demonstrated the importance of the type of structural defects in the contact area of b-SWCNTs on the contact resistance of the T-junction of SWCNTs in the resulting electrical conductivity of the final network [42]. A layered design was proposed using natural polymer matrices, i.e., collagen, albumin, or chitosan, connected via the b-CNTs to provide an electrically conductive network to assist cells in their mutual reconnection during the regenerative process [42].

Finally, a bio-inspired design was proposed for b-CNTs as nanocomposite reinforcement. Coarse-grained MD simulations revealed that the pullout strength of the b-CNTs could be an order of magnitude higher than that of linear CNTs, whereby the enhanced interfacial shearing strength was found to be strongly dependent on various parameters, such as the geometry of nanofibers, the molecular weight of the polymers composing the bulk material, and the pullout velocity [3].

## 3. Applications

In recent years, there have been many proposed applications in materials technology utilizing various types of branched CNSs, resulting from their interesting mechanical, thermal, electrical, and electronic properties. Application areas of major interest include battery electrodes (Section 3.1, Table 1), electro-catalysis (Section 3.2, Table 2), super-capacitors (Section 3.3, Table 3), electromagnetic wave (EMW) shielding (Section 3.4, Table 4), sensors (Section 3.5, Table 5), and composite performance enhancement (Section 3.6).

### 3.1. Batteries

**Figure 3:**
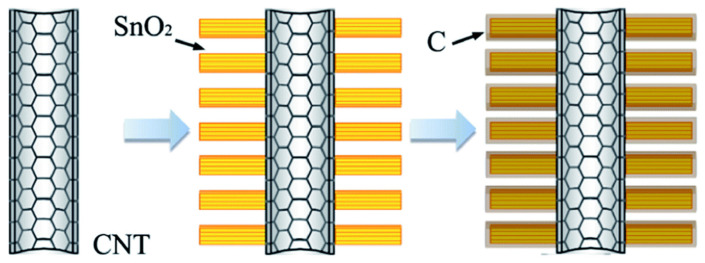
Schematic illustration of the formation of the branched MWCNT@SnO_2_ nanorod@carbon heterostructures. Used with permission of The Royal Society of Chemistry from [44], Copyright© 2013; permission conveyed through Copyright Clearance Center, Inc.

**Figure 4:**
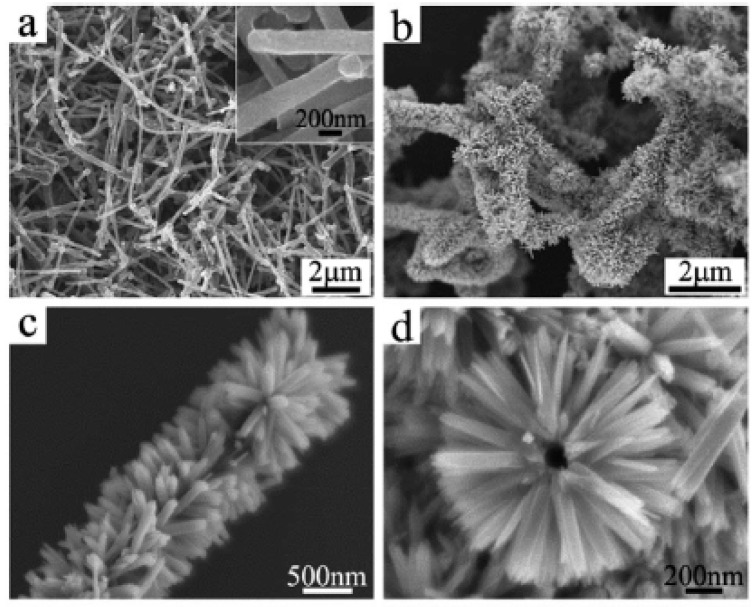
SEM images of b-CNTs (**a**) and b-CNT@SnO_2_ heterostructures (**b**–**d**). Used with permission of The Royal Society of Chemistry, from [44], Copyright© 2013; permission conveyed through Copyright Clearance Center, Inc.

Lithium-ion batteries (LIBs) use oxides of the valuable elements Li, Co, Ni, and Mn in their cathode material. This accounts for about a third of the cost of the battery. However, cheaper technologies may not be commercially recyclable. Therefore, the main way to reduce their carbon footprint would be to make them more efficient, so that they are not only cheaper to begin with, but also last longer in service. LIBs mainly use graphitic materials in their anodes as they have good electrical conductivity, high crystallinity, and a layered structure. Graphite has a theoretical intercalation capacity of 372 mA·h·g^−1^ for the end compound LiC_6_ [43]. LIB anodes fabricated with branched CNSs (Table 1) together with transition metal sulfide or oxide NPs have high capacity and good cycle life [44,45,46,47,48,49]. The synergy of these NPs and branched CNSs leads to a hierarchical porous network with a large surface area, good electrical conductivity, and enhanced structural stability compared to conventional amorphous carbon. As an example, Chen et al. fabricated a b-CNT@SnO_2_@carbon sandwich-type heterostructure (Figure 3) as an LIB anode [44]. Scanning electron microscopy (SEM) images (Figure 4) confirmed the branched nature of CNTs (Figure 4a), which increased significantly in diameter upon inclusion of SnO_2_ in the structure (Figure 4b), which featured a “brush-like” morphology, with SnO_2_ nanorods stemming from CNTs (Figure 4c,d). Hierarchical architectures were attained also through iron oxide NP encapsulation inside CNTs, whose 3D networks were ozonized and used as substrates to further grow CNT branches, thanks to a catalytic effect of the iron NPs. This material showed high stability (>98% capacity retention up to 200 cycles at 100 mA·g^−1^ with a coulombic efficiency >97%, an outstanding rate capability (>70% capacity retention at 50–1000 mA·g^−1^ rates), and reasonable capacity of ca. 800 mA·h·g^−1^ at 50 mA·g^−1^ [50].

Sodium-ion batteries (SIBs) have the advantage that sodium is cheaper and more abundant than lithium. However, sodium ions are much larger than lithium ions, so new electrode materials are being investigated. SIBs do not use graphite, as it is not possible to achieve sodium insertion with the electrolytes commonly used in metal-ion batteries. In theory, 2D carbon materials can have a theoretical intercalation capacity up to 2232 mA·h·g^−1^ for the end compound Na_6_C_6_ [51]. In practice, branched CNSs are amongst the most promising anode materials for SIBs (Table 1) as they have good conductivity and a 2D/3D micro-porous structure. The structure of b-CNT anode material means that the sodium-ion or lithium-ion insertion follows the “house of cards” model [52], which means that both sides of the branches are accessible. These types of anode materials are intrinsically flexible, and so the mechanical stresses on intercalation/de-intercalation are reduced, which leads to greater electrode long-cycle stability. Branched CNSs serve as conductive porous networks with the active NPs, and they not only improve mechanical stability but also enhance the surface area [49,53,54].

**Table 1:** Examples of branched CNSs used in battery technology.

CNS Type	Electrode Type	Specific Capacity	Ref.
Y-type. Nitrogen-doped porous branched MWCNTs (NBNTs)	Co_9_S_8_@NBNT ^1^ nanohybrid anode, Li metal cathode-coin cell	1109 mA·h·g^−1^ at 500 mA·g^−1^ current density (200 cycles)	[45]
Tree-like bud-branch composite. VS_2_ nanosheet “buds” on MWCNT “trees”	VS_2_ NS@CNTs ^1^ film on Cu foil current collector as anode working electrode, Li metal foil as counter electrode	~850 mA·h·g^−1^ at 200 mA·g^−1^ current density (100 cycles)	[46]
Porous 3D interconnected network of CNS and NiO nanofibers	CNS/NiO coated on Cu foil current collector anode as a working electrode and lithium foil as reference and counter electrode	~750 mA·h·g^−1^ at 200 mA·g^−1^ current density (50 cycles)	[47]
Tree-like. MWCNTs with SnO_2_ branches coated with carbon—“brush-like” structure	b-CNT@SnO_2_@C ^1^ Heterostructures as anode	~800 mA·h·g^−1^ at 720 mA·g^−1^ current density (40 cycles)	[44]
Tree-like. MWCNT truck with Bi_2_S_3_ nanorod branches—“brush-like” structure	Bi_2_S_3_-CNT branched hybrid anode with Li metal foil as counter and reference electrode	~400 mA·h·g^−1^ at 60 mA·g^−1^ current density (40 cycles)	[48]
Porous carbon hybrids (PAN and MWCNTs)—metal-based nanostructures (MOF)	Co-carbon hybrids (CoCHs) as anodes for rechargeable metal ion batteries	LIB: 680 mA·h·g^−1^ at 200 mA·g^−1^ current density (320 cycles) SIB: 220 mA·h·g^−1^ at 100 mA·g^−1^ current density (500 cycles)	[49]
Core/branch cocoon of MWCNT on sodium manganite nanotubes	SIB cathode material	158 mA·h·g^−1^ at 100 mA·g^−1^ current density (100 cycles)	[53]
Tree-like. Graphene foam supported V_2_O_3_/MWCNTs core/branch composite arrays	SIB cathode material	612 mA·h·g^−1^ at 100 mA·g^−1^ current density (70 cycles)	[54]

^1^ @ denotes core@shell structure.

### 3.2. Electrocatalysis

CNSs have been widely studied in electrocatalysis, usually in combination with other components, such as metals [55,56,57,58,59,60,61,62,63,64] and oxides [65,66,67,68,69,70,71,72] or both [73,74,75,76,77], to attain enhanced performance [78], but also in metal-free electrocatalysis [79] for more sustainable solutions [80]. Branched CNSs are promising materials to use with various electrocatalysts for cathodic oxygen reduction reaction (ORR) [81,82,83,84,85,86], oxygen evolution reaction (OER) [81,84,87,88], and hydrogen evolution reaction (HER) [89,90,91,92]. Applications include fuel cells, metal–air batteries, and other electrochemical energy conversion and storage systems (Table 2). The branched nanostructure can be very advantageous, as described for the case of proton-exchange membrane fuel cells, whereby branching of CNTs onto clay nanoplatelets provided ideal nanofillers for composites with excellent water diffusion behavior and very high proton conductivity in drastic conditions, both of temperature and humidity [93].

**Figure 5:**
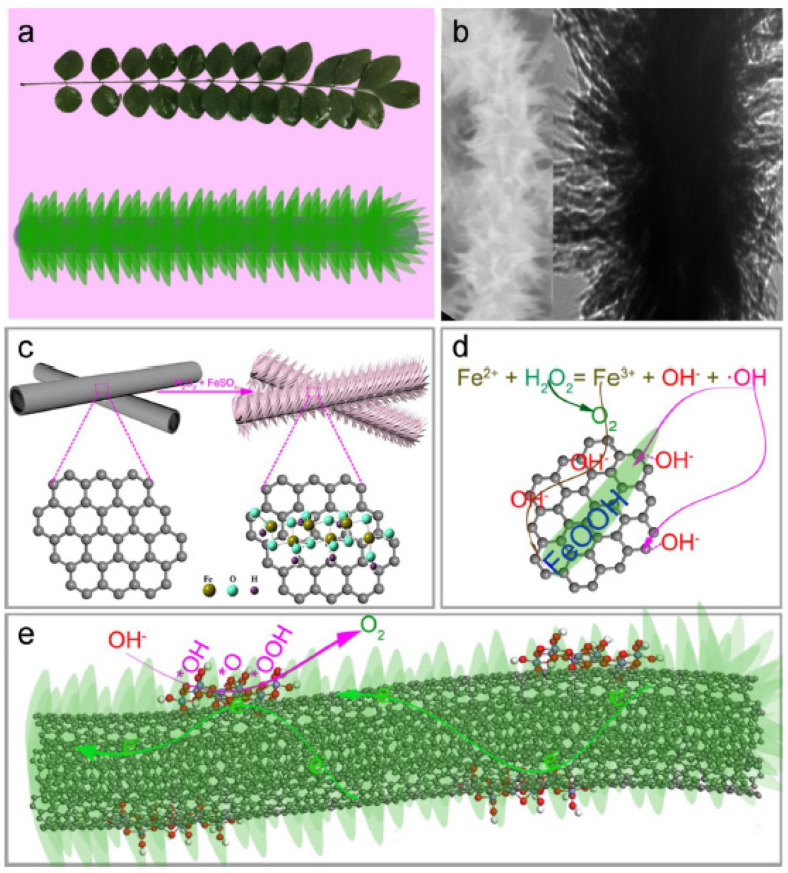
(**a**) Model of a leaf–branch structure; (**b**) electrocatalyst with a leaf–branch structure; (**c**) the leaf–branch synthesis process, and (**d**) reaction mechanism of the electrocatalyst. (**e**) Schematic of FeOOH@CNTs electrocatalysis process. Reprinted from [87], Copyright© 2019, with permission from Elsevier.

The many advantages of branched CNSs in electrocatalysis have been analyzed in detail in many studies. For instance, Li et al. fabricated a tree-like nanostructure with FeOOH leaves growing on MWCNT branches, FeOOH@MWCNTs, as an anode for electrocatalytic water splitting (Figure 5) [87]. Another work combined the catalytic activity of cobalt NPs with the high conductivity of CNT branches grown onto reduced graphene oxide (rGO), which displays a high surface area and ion diffusion, to produce a hierarchical architecture for electrocatalytic ORR and OER reactions [94]. Bamboo-like branches of CNTs were grown onto rGO, thanks also to iron oxide NPs, to enhance the performance of LIBs and achieve a specific capacity as high as 1757 mA·h·g^−1^ at 50 mA·g^−1^, with a good rate capability of 73% at 1000 mA·g^−1^, and a gradual increase from ca. 1500 to ca. 2900 mA·h·g^−1^ after 100 cycles [95].

**Table 2:** Recent examples of branched CNSs used in electrocatalysis.

CNS Type	Application	Specifications	Ref.
Biomimetic tree-like, bud-branching	Oxidized MWCNTs with FeOOH “leaves” on Ni foam anode—OER electrocatalyst for water splitting	OER overpotential 210 mV at 10 mA·cm^−2^. Tafel slope of 31 mV·dec^−1^	[87]
3D macroporous. Hierarchical graphene/iron hybrid architectures branched by *N*-doped MWCNTs	Iron oxide decorated *N*-doped MWCNTs and iron oxide decorated MWCNTs grown on rGO ^1^ to form hybrid structures for bifunctional electrocatalysis	ORR onset potential 0.72 V, OER onset potential 1.63 V, Tafel slope of 61 mV·dec^−1^	[82]
Tree-like hierarchical network	Fe/*N*-doped MWCNTs with Fe/*N*-doped b-CNT-ORR electrocatalyst in proton exchange fuel cells (PEMFCs)	ORR onset potential ~0.92 V, Tafel slope of ~60 mV·dec^−1^	[83]
Tree-like hierarchical architecture	Ni_3_Co NP catalysis to form *N*-doped MWCNT branches on CNFs-electrocatalyst for hydrogen production via water splitting	HER overpotential 114 mV at 10 mA·cm^−2^, Tafel slope of 117 mV·dec^−1^	[89]
3D non-aligned hierarchical network	Fe catalyzed growth of primary and secondary branching MWCNTs on a glassy carbon (GC) substrate, then Pt NP electrodeposition to form electrocatalyst	High activity and poisoning stability of the Pt-CNT/CNT/GC electrodes for MeOH oxidation	[96]
3D flower-like structure	N, P co-doped MWCNTs using multi-armed ZIF-8 templating for ORR, OER, and metal-ion batteries	ORR onset potential ~0.75 V, OER onset potential ~1.5 V, Tafel slope of 115 mV·dec^−1^	[84]
Tree-like, P-doped MWCNT with amorphous MoS_2_ leaves	Urea-assisted hydrothermal synthesis of tree-like MoS_2_/MWCNTs composite—HER electrocatalyst	HER overpotential 151 mV at 10 mA cm^−2^, Tafel slope of 49 mV·dec^−1^	[90]
Tree-like MWCNTs with MoS_2_ flake leaves	Leaves-and-branch structure of strongly coupled and porous MoS_2_-MWCNTs nanocomposite—HER electrocatalyst	HER overpotential ~100 mV at 10 mA·cm^−2^, Tafel slope of 47 mV·dec^−1^	[91]
3D hyperbranched structure	Dendritic hyperbranched HPEK grafted onto the surface of MWCNTs and sulfonated to get water-dispersible SHPEK-g-MWCNT. ORR electrocatalyst.	ORR onset potential ~0.22 V	[85]

^1^ rGO = reduced graphene oxide.

### 3.3. Supercapacitors

Branched CNSs are not only used in conventional energy storage systems, such as LIBs and fuel cells, but also as promising materials for supercapacitors [97]. The term supercapacitor encompasses electrostatic double-layer capacitors (EDLCs) [98,99,100,101] and pseudocapacitors [102,103,104]. Carbon-based electrodes tend to be EDLCs, as their porosity lies in the microporous (>2 nm) to mesoporous (2–50 nm) range. In EDLCs, the separation of charge in a Helmholtz double-layer is ca. 0.3–0.8 nm at the interface between the electrode surface and the electrolyte. There is no transfer of charge between the electrode and the electrolyte. The forces leading to the polarization of absorbed molecules are electrostatic; therefore, CNSs are not chemically modified.

**Figure 6:**
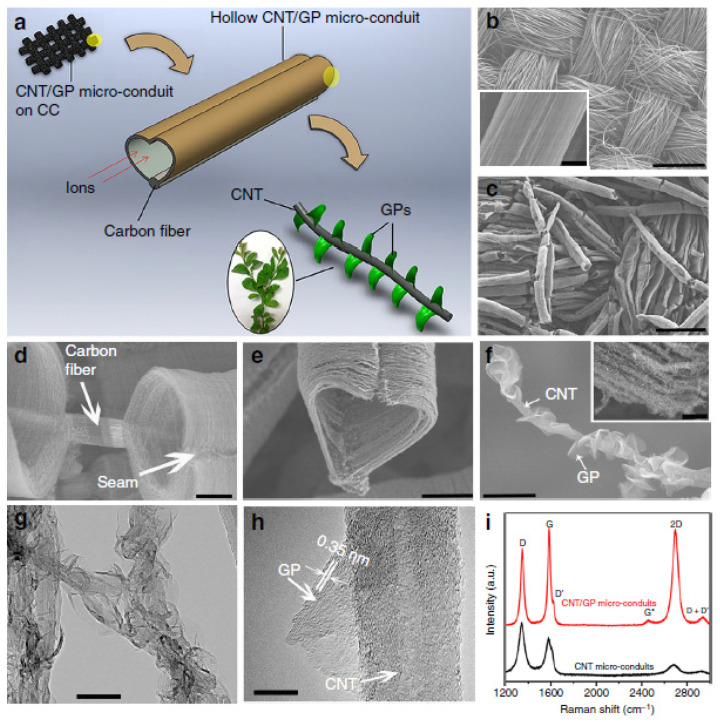
Structural characterization of CNT/GP micro-conduits. (**a**) Schematic illustration of CNT/GP micro-conduits in a leaves-on-branchlet nanostructure on CC substrates for high-performance supercapacitor electrodes (note that the yellow shaded areas in the schematic indicate the selected areas to be magnified). (**b**) Bare CC substrate at low magnification (inset shows the surface of a single carbon fiber). (**c**) Uniform coverage of CNT micro-conduits on carbon fibers at low magnification. (**d**) A close-up of CNT micro-conduits on a carbon microfiber. (**e**) A CNT/GP micro-conduit in a heart shape. (**f**) A single CNT decorated with many GPs at high magnification (inset shows GPs on CNT micro-conduit array walls). (**g**) TEM image of the hierarchical structure. (**h**) HR-TEM image of a petal emerging from a nanotube. (**i**) Comparative Raman spectra of CNT micro-conduits and CNT/GP micro-conduits on CC substrates. Scale bars: (**b**) 500 μm (inset: 3 μm), (**c**) 300 μm, (**d**) 10 μm, (**e**) 20 μm, (**f**) 300 nm (inset: 2 μm), (**g**) 100 nm, (**h**) 10 nm. Reproduced from [99] under a Creative Commons license http://creativecommons.org/licenses/by/4.0/ (accessed on 7 October 2021).

Table 3 shows examples of branched CNSs with applications in supercapacitor technology. They have a very large surface area and excellent electrical conductivity, which leads to high power and energy density materials. For instance, Xiong et al. have fabricated a MWCNT tree-like hybrid nanostructure with graphite platelet (GP) leaves for supercapacitor applications (Figure 6) [99].

**Table 3:** Examples of branched CNSs used in supercapacitor technology.

CNS Type	Material Type	Specifications	Ref.
Tree-like, bud-branch composite	MWCNTs grown on MoO_2_ NPs decorating Mo-O-C nanorods for pseudocapacitive energy storage	Specific capacitance of 1667 F·g^−1^ at 1 A·g^−1^ discharge rate. Rate capability of 93% after 3000 cycles (5 A·g^−1^)	[103]
Tree-like MWCNTs with carbon films	Vertically aligned CNTs on stainless steel substrate for electrochemical capacitor electrodes	Areal capacitance of 0.55 mF·cm^−2^ (4.6 F·cm^−3^) at a current density of 0.88 mA·cm^−2^ (2500 cycles)	[100]
Y-type branched MWCNT/CNF	b-CNT/b-CNF composite for supercapacitors	Specific capacitance of ~207 F·g^−1^ at 1 A·g^−1^ discharge rate. Rate capability of 96% after 5000 cycles (20 A·g^−1^)	[101]
3D structure composed of MWCNT tree-like with CNF leaves	Hollow MWCNT/GP micro-conduits composed of MWCNTs with GP branchlets for supercapacitor electrodes	Specific capacitance of 500 F·g^−1^. Areal capacitance of 2.35 F·cm^−2^. Rate capability of ~95% after 10,000 cycles	[99]
3D core–shell branched nanostructure CNTs/Ni(OH)_2_ composites	3D branched MWCNTs/Ni(OH)_2_ as a positive electrode for battery–supercapacitor hybrid device (BSH)	MWCNT/Ni(OH)_2_ cathode-specific capacitance of 1251 F·g^−1^ at 1 A·g^−1^. Rate capability of 75% after 2000 cycles	[104]

### 3.4. Electromagnetic Wave (EMW) Technology

**Figure 7:**
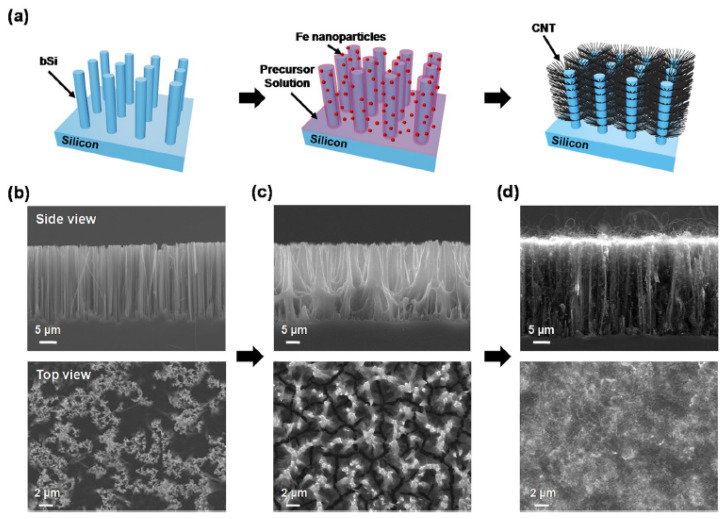
(**a**) Three-dimensional schematic illustration of the fabrication process for the bSi-CNT structure. (**b**–**d**) SEM images showing side views (top panels) and top views (bottom panels) of the bSi-CNT sample corresponding to (**a**). Reproduced from [105], under a Creative Commons license http://creativecommons.org/licenses/by/4.0/ (accessed on 7 October 2021).

Branched CNSs are used to enhance EMW absorbance (Table 4), which has promising applications in the area of photovoltaics (PV), photo-detectors, and water splitting by photo-electrochemical catalysis. Phan and Yu fabricated tree-like vertically aligned nanostructures (VANS) with MWCNT branches on black silicon (BSi) stems for EMW absorbance applications (Figure 7) [105].

**Table 4:** Examples of branched CNSs used in EMW technology.

CNS Type	Material Type	Specifications	Ref.
3D tree-like hybrid. VANS (vertically aligned nanostructure)	MWCNTs grown from iron catalysts on black-Si stems, vertically aligned on Si substrate; ultrahigh absorbance at wide spectral range of wavelength	Absorbance of bSi-CNT in the range 300–1200 nm and was 94% at 1200 nm	[105]
Flower-like hierarchical nanospheres	Fe catalyzed growth of primary and secondary branching MWCNTs on a glassy carbon substrate; electromagnetic wave absorbers	Reflection loss is −50 dB at a frequency of 7.9 GHz, and efficient absorption bandwidth of 4 GHz	[106]
Tree-like. Hollow porous carbon fibers (HPCFs) with MWCNT branches decorated with iron oxide NPs	Fe_3_O_4_–CNTs–HPCF absorbents with “tree-like” structure; EM wave absorber. Lightweight composite material for aerospace applications	The bandwidth with a reflection loss <−15 dB from 10 to 18 GHz (1.5–3.0 mm thick layer) and, the minimum reflection loss is −51 dB at 14 GHz (2.5 mm thick layer)	[107]

### 3.5. Sensors Technology

**Figure 8:**
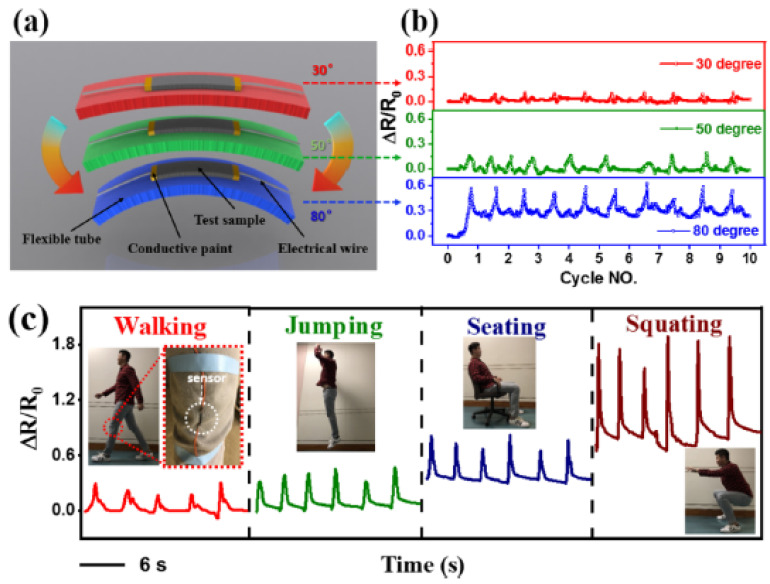
(**a**) Schematic of the 0.7 TPU/CNS test sample under bending with different bending degrees; (**b**) corresponding electrical signal of ΔR/R_0_ plotted as a function of cycle number under 30°, 50°, and 80° bending. (**c**) Response of a sensor to different human motion patterns (e.g., walking, jumping, seating, and squatting) when the sensor was mounted on the knee joint. Reprinted with permission from [108], Copyright© 2018, American Chemical Society.

Branched CNS fillers enhance the electrical properties of thermoplastic polyurethane (TPU) as they have a low percolation threshold and good electrical conductivity. Application areas include strain sensors and actuators [108,109,110]. In the application area of strain sensors, researchers usually report a gauge factor (G_f_), calculated from the ratio of normalized instantaneous resistance change (ΔR/R_0_) to strain (ε). Other sensor applications include highly sensitive electrochemical sensors in so-called nanohybrid sensors [111,112,113,114]. Manas-Zloczower et al. fabricated highly stretchable stain sensors with carbon nanostructures (CNS supplied by Applied Nanostructured Solutions LLC) in a thermoplastic polyurethane (TPU) matrix (Figure 8) [108].

**Table 5:** Examples of branched CNSs used in sensing.

CNS Type	Material Type	Specifications	Ref.
Forked-branched hybrid network CNS	3D CNS decorated with MWCNTs or carbon black-TPU-based composite piezoresistive strain sensors	Composite 1.5CNS-1CB-gauge factor ~21 (strain ε = 50%) and 99 (strain ε = 100%)	[109]
Branched CNS-highly-entangled and wall-sharing MWCNTs	Branched CNSs in TPU matrix as stretchable strain sensors	Electrical percolation threshold (ΦC) of 0.82 wt%. Gauge factors of 15, 30, and 58 for strain levels of 0–44%, 45–73%, and 74–100%, respectively	[110]
CNFs with GNP leaves	TPU melt-mixed with MWCNTs and GNPs-dielectric elastomers for shape memory and temperature sensing	An increase in dielectric constant of 10 with low loss tangent, 0.008. Dielectric constant of 13 at RT (1 kHz)	[111]
Tree-like MWCNT with CNS leaves	Branched CNS in TPU-highly stretchable strain sensors	Percolation threshold of 0.06 wt% CNS. Composite with 0.7 wt% CNS; electrical conductivity of 1 S/m; gauge factor up to 6861 at strain ε = 660% (elongation at break is 950%)	[108]
Y-junction MWCNTs and carbon flakes	TPU/CNS/GNP nanocomposites—melt mix preparation—multifunctional polymer nanocomposites, piezoresistive sensors	At 2 wt% filler concentration: TPU/CNS and TPU/CNS/GNP nanocomposites; gauge factor up to 28 and 144, respectively, under 50% strain	[112]
Tree-like MWCNT-COOH/Ag NP with porous structure	MWCNT-COOH/Ag NP nanohybrid CO_2_ gas detection	Electrochemical CO_2_ detection in aqueous medium with a detection limit of 52 nM (surface area 525 ms/g)	[113]
Hierarchically structured carbon electrodes fabricated from cellulose	MWCNT modified carbon fiber: single fiber microelectrode with branched carbon nanotubes for enhanced sensing	Tested the detection of NADH oxidation. The overpotential of NADH decreased from over 0.8 V to 0.6 V for the CNT-modified carbon fiber electrode	[114]

### 3.6. Composite Performance Enhancement

**Figure 9:**
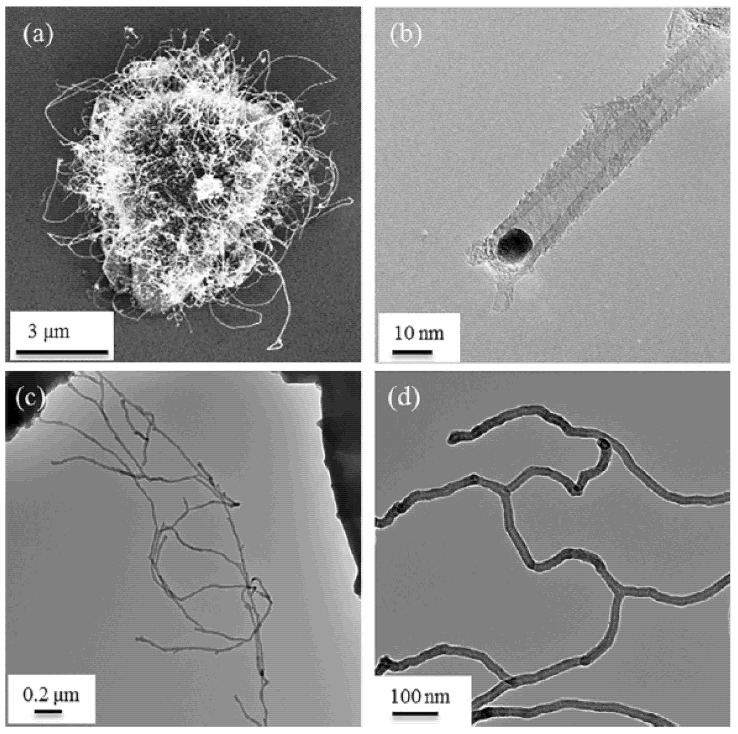
Micrographs of the magnetite NPs and formed b-MWNTs: (**a**) SEM overview of CNTs growing from pumice; (**b**) TEM detail of nucleating Fe_3_O_4_ NP; (**c**) TEM overview of b-MWNTs; (**d**) TEM detail of b-MWNTs. Reproduced from [116], Copyright© 2018, with permission from Elsevier.

Researchers have found that branched CNSs significantly enhance the mechanical, electrical, and thermo-conductive properties in a wide range of nanocomposites. Branched MWCNT fillers form stronger networks by comparison with conventional MWCNTs, leading to enhanced mechanical performance of the resulting materials [13,19,21,115,116,117,118]. Natural catalysts can be used to fabricate branched MWCNTs as reinforcement materials to enhance the mechanical and electrical properties of composites. This was shown with volcanic pumice from the Greek island of Santorini (Figure 9) [116]. Alternatively, hyperbranched polymers can be grafted onto CNTs to favor their interconnection and interfacing with polymer resins, with additional advantages such as lower temperatures for curing [20].

The presence of branched as opposed to linear CNTs can be advantageous for the mechanical properties. For instance, the branched structure allows for a lower rheological percolation threshold at much lower nanofiller content, as shown on thermoplastic polyurethane (TPU) composites that demonstrated enhanced conductive and mechanical properties upon inclusion of b-CNTs as fillers [115].

Using a different strategy, hyperbranched polyesters were grafted onto the surface of carboxylated MWCNTs through an esterification reaction. When included in epoxy resins, the fillers demonstrated stronger interfacial bonding and toughening performance, with higher degree of branching of the polymer [19]. Another hyper-branched polymer used to connect MWCNTs is poly(urea–urethane) so as to provide fillers for polyamide-6. The grafted polymer enhanced the compatibility between the CNTs and the bulk matrix of the composite, enabling hydrogen bonding between them, thus yielding a uniform dispersion and allowing for more efficient load-transfer from the polyamide to the CNTs, as well as lower crystallization temperature and higher crystallization rate and degree [21].

Compression-molded samples were prepared with different polymers (i.e., polypropylene (PP), polycarbonate (PC), and poly(vinylidene fluoride) or PVDF) and different CNT fillers (i.e., SWCNTs, linear or branched MWCNTs). The nanofillers enhanced the conductive properties of the polymers, allowing for a lower electrical percolation threshold already with <0.1% CNT content. Furthermore, inclusion of 2 wt% b-MWCNTs led to resistivity as low as 2 Ω cm (PC), 3 Ω cm (PVDF), or 7 Ω cm (PP). Another additional advantage of b-CNTs is the high homogeneity of the resulting dispersions [117].

### 3.7. Other Technological Applications

**Figure 10:**
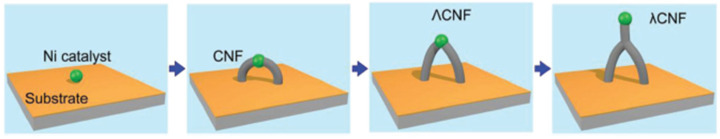
Schematic growth of lambda-shaped CNFs (not to scale). Reproduced with permission from [122], © 2019 WILEY-VCH Verlag GmbH & Co. KGaA, Weinheim.

**Figure 11:**
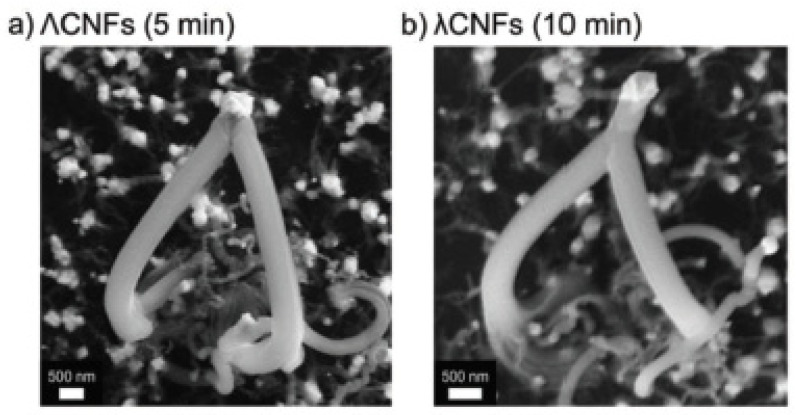
(**a**) SEM images of ΛCNF after growth times of 5 min. (**b**) Increasing the growth time leads to λCNFs. These samples grow after 10 min. The catalytic centers are visible as bright particles at the ends of the lambda-shaped CNFs and consist presumably of nickel. Reproduced with permission from [122], © 2019 WILEY-VCH Verlag GmbH & Co. KGaA, Weinheim.

Other technological applications for branched CNSs take advantage of aspects of their tunable properties, such as rheology [119] and hydrophobicity [5], as well as their electrical and electronic properties [8,16,18,29,120,121]. Hirtz and Hölscher et al. used an open flame process to fabricate site-specific catalyst supports and also proposed an empirical model for the growth process, as shown in Figure 10, in agreement with SEM micrographs (Figure 11) [122].

A nanothickening agent for high temperature fracturing fluid in the field of oil and gas production was produced using dendritic structures obtained from the free radical polymerization of acrylamide (AM), acrylic acid (AA), sodium *p*-styrene sulfonate, dimethyl diallyl ammonium chloride, and MWCNTs. The resulting system demonstrated enhanced thickening capacity, thermoresistance, salt and shear tolerance, viscoelasticity, sand carrying capacity, and gel breaking performance, compared to the pristine polymer and partially hydrolyzed polyacrylamide (HPAM) [119].

Super-hydrophobicity is a desirable property that finds various applications, such as the production of stain proof and advanced textiles. This property can be achieved through the creation of highly rough surfaces, for instance through a biomimicry approach that take inspiration from the microstructure of the lotus leaf, which floats on muddy waters whilst appearing clean. In the case of branched CNTs, lotus-leaf mimicry can be attained in different ways. In one approach, the CNTs were vertically aligned and branched, so as to create a tree-like structure with extreme non-sticking properties and a water contact angle as high as 165° [5]. Alternatively, sidewall functionalization of MWCNTs with branched and linear perfluoropolyether (PFPE) was achieved through the generation of reactive radical species from the thermal decomposition of PFPE peroxide precursors. The wettability of the functionalized MWCNTs was significantly reduced, as they acquired super-hydrophobicity [120].

Water purification is another field of potential application for branched CNSs. Purification membranes were obtained through the electrospinning of chitosan fibers and concomitant spraying of carboxylated MWCNTs, and successfully applied for the removal of methylene blue and methyl orange dyes, also with a water flux as high as 3757.36 L/m^2^h, and at a low pressure of 0.2 bar. In this manner, an alternative production method to the common blending process was demonstrated to be useful in wastewater treatment [121].

## 4. Conclusions and Future Perspectives

The European Green Deal aims to make Europe climate neutral by 2050, boosting the economy through green technology, creating sustainable industry and transport, and cutting pollution [123]. This means that the commercial success of any of the applications highlighted in this concise review has to be not only scalable but also sustainable. As detailed in this work, both theory and experiments have shown that branched MWCNTs can enhance the mechanical and electrical properties of polymeric materials. One of the advantages of these nanostructures is that the outer layers are branched and so may have degraded properties, but the inner layers are intact and retain desired properties. Further studies to compare the mechanical properties between the different types of junctions and unbranched CNTs are needed. At present, in 2021, SWCNTs have been studied in silico and unsurprisingly revealed a superior mechanical performance relative to branched SWCNTs, with structural damage initiation occurring at non-hexagonal sites, although with various failure modes and strength reduction depending on the exact bonding structure at the junction [124]. In addition to composites’ reinforcement, the area of catalysis presents some interesting opportunities. Branched MWCNTs are used, or have proposed applications, as catalyst support materials, both for noble-metal catalysts such as Pt as well as earth abundant catalysts such as Fe and Ni, and even on their own in metal-free electrocatalysis.

The most commonly used fabrication method in this field is some type of CVD, so the produced material would have to have unique properties to be very attractive to a commercial enterprise. Therefore, new production methods that use sustainable, natural resources and are highly efficient are continuously sought after, also to produce branched CNSs for additional advantages, such as the possibility to avoid polymer binders in devices for electrochemical energy storage and conversion, so as to avoid the inclusion of additional components that could increase the resistance and negatively affect the device performance [125]. Understanding the CNT growth mechanisms in detail is key to allowing a leap forward to overcome current critical challenges for their mainstream commercial application [126]. For instance, entirely end-bonded MWCNTs were found to exhibit superconductivity with a transition temperature that was significantly higher than that of ropes of SWCNTs, and such a property is highly sensitive to the CNT junction structure [127]. Furthermore, CNT growth in the CVD chamber is non-linearly dependent with reaction time, and fine adjustment of experimental parameters to maximize the catalyst performance is key to ensure high-quality CNT vertical forests [128,129]. CNT orientation is an important parameter in the determination of the final material physicochemical properties, with CNT–CNT junctions playing a key role [130].

Considering the great research efforts required over the last decades to understand how to control unbranched CNT growth and anisotropic orientation, the translation of such knowledge onto b-CNTs to attain fine control over their production presents further challenges that are currently limiting their large scale use. Availability of commercial suppliers that offer high-grade b-CNTs with defined structures and good batch-to-batch reproducibility is still limited, as is the current market demand that could promote its progress. Clearly, more advances are needed at the fundamental-research level to drive b-CNT development and adoption.

Finally, researchers in this field can take hope from Herbert Krömer (Nobel Prize in Physics 2000) in Krömer’s Lemma: “The principal applications of any sufficiently new and innovative technology always have been and will continue to be applications created by that new technology” [131]. For instance, the unique properties of CNS led to a wide array of proposed applications in medicine and the health sector [132,133,134,135], spanning from tissue engineering [35,136] to wearable sensors [137,138], imaging [139,140], diagnostics, and therapy [141], especially leveraging their conductive properties [142]. There are, however, hurdles for their translation into clinical practice [143,144,145], and it can be envisaged that further developments in their covalent attachment into stable and branched nanostructures may allow at least some of these barriers to be overcome, for instance limiting the loss of individual CNSs from the material.

## Data Availability

Not applicable.
